# Concussions in young adult athletes: No effect on cerebral white matter

**DOI:** 10.3389/fnhum.2023.1113971

**Published:** 2023-03-01

**Authors:** Linda J. Hoffman, Rachel E. Mis, Caroline Brough, Servio Ramirez, Dianne Langford, Tania Giovannetti, Ingrid R. Olson

**Affiliations:** ^1^Department of Psychology and Neuroscience, Temple University, Philadelphia, PA, United States; ^2^Lewis Katz School of Medicine, Temple University, Philadelphia, PA, United States

**Keywords:** concussion, white matter, axonal shearing, sports-related concussion, diffusion weighted imaging

## Abstract

**Introduction:**

The media’s recent focus on possible negative health outcomes following sports- related concussion has increased awareness as well as anxiety among parents and athletes. However, the literature on concussion outcomes is equivocal and limited by a variety of diagnostic approaches.

**Methods:**

The current study used a rigorous, open- access concussion identification method—the Ohio State University Traumatic Brain Injury Identification method (OSU TBI-ID) to identify concussion and periods of repeated, subclinical head trauma in 108 young adult athletes who also underwent a comprehensive protocol of cognitive tests, mood/anxiety questionnaires, and high-angular-resolution diffusion-weighted brain imaging to evaluate potential changes in white matter microstructure.

**Results:**

Analyses showed that athletes with a history of repetitive, subclinical impacts to the head performed slightly worse on a measure of inhibitory impulse control and had more anxiety symptoms compared to those who never sustained any type of head injury but were otherwise the same as athletes with no history of concussion. Importantly, there were no group differences in cerebral white matter as measured by tract- based spatial statistics (TBSS), nor were there any associations between OSU TBI-ID measures and whole-brain principal scalars and free-water corrected scalars.

**Discussion:**

Our results provide support for the hypothesis that it is not concussion per se, but repetitive head impacts that beget worse outcomes.

## 1. Introduction

Sports-related concussion (SRC) is a common injury that has been gaining heightened publicity in recent years due to fears surrounding the long-term consequences of repeated, brain injury (Frieden et al., 2015; Baldwin et al., 2018). As of Harmon et al. (2013), the estimated annual incidence of sports-related concussion in the United States alone was 3.8 million. Participation in athletic pursuits increases one’s chances of sustaining a concussion compared to the general populace by a factor of 50 (Wilberger et al., 2006). Media attention to this problem has caused parents to become increasingly worried about allowing their children to engage in sports with a high incidence of concussion such as American football. This is reflected in the decreasing number of youths enrolled in youth football (Findler, 2015).

Accumulating evidence shows an association between *repetitive* head trauma, such as that experienced among professional athletes in contact sports like American football, and neurodegeneration accompanied by mood changes and cognitive decline (i.e., chronic traumatic encephalopathy CTE; Asken et al., 2017). Do these findings generalize to young adult athletes who might experience periods of repetitive subclinical impacts to the head? Or is the sustainment of one or multiple concussions enough to beget similar impairments?

The pathophysiology of concussion involves disruption of cerebral white matter (Weber et al., 2018; Hellewell et al., 2020). This can be examined *in vivo* by measuring changes in signal from diffusion weighted imaging (DWI) metrics. Our review of the existing literature on non-professional athletes in the chronic stage of concussion found a surprising lack of consensus about changes in cerebral white matter. For example, two studies found instances of *higher* fractional anisotropy (FA) in athletes with a history of concussion versus those without a history of concussion in some distinct white matter tracts (Churchill et al., 2019), while another study found differences in a different set of white matter tracts (Churchill et al., 2017). Meanwhile, a different study reported that athletes with concussion histories have relatively *lower* FA compared to controls (Tremblay et al., 2014), while several other studies have reported no changes in FA anywhere in the brain (List et al., 2015; Chamard et al., 2016; Meier et al., 2016a).

This mix of findings also manifests when other DWI white matter metrics are examined. For example, some studies report widespread instances of lower radial diffusivity (RD; Sasaki et al., 2014; Wright et al., 2021), some report higher RD (Koerte et al., 2012; Murugavel et al., 2014; Cubon et al., 2018), and some report no differences (see also Pasternak et al., 2014; Tremblay et al., 2014; Chamard et al., 2016; Clough et al., 2018; Mustafi et al., 2018). Meanwhile, some chronic studies have reported concussion-related decreases in axial diffusivity (AD) in the corpus callosum when comparing those with multiple concussion to those with one concussion (Chamard et al., 2016). Meanwhile, other studies of former athletes find no changes in AD between those with and without a history of concussion (Tremblay et al., 2014; Mustafi et al., 2018; Caron et al., 2020; Wu et al., 2020).

In sum, there is no clear consensus regarding the effect of history of concussion(s) on white matter in non-professional athletes (Slobounov et al., 2012; Asken et al., 2017).

In our empirical study we were motivated to contribute to a foundation of transparency and replicability in the concussion literature by operationalizing retrospective concussion status by using the Ohio State University Traumatic Brain Injury-Identification (OSU TBI-ID), as other studies of a retrospective nature have failed to do so in the past. We further broke down our sample for two separate sets of comparisons. The first sample breakdown consisted of three groups, comprised of those with no history of concussion (NCHx), those with a single life-time concussive event (SCHx), and those who sustained multiple concussions over the course of their lifetime (MCHx). The second breakdown consisted of all of the aforementioned groups, only with those who sustained periods of repeated, subclinical impacts to the head factored out and into their own group (RHx). The current study also aims to identify the potential provenance of any microstructural and behavioral relations by looking at every possible OSU-TBI ID variable (i.e., total number of concussions, number of losses of consciousness, time since last injury, age at first injury, age at onset of repetitive trauma, duration of repetitive trauma) in a continuous fashion, as well as by interrogating potential confounding variables that have an impact on cognition and are known to have an association with concussion (i.e., depression, anxiety, etc.). To examine neural changes, we used high-angular-resolution DWI to measure white matter (Mueller et al., 2015).

## 2. Materials and methods

### 2.1. Participants

The study included 108 18- to 33-year-old athletes from the Temple University student population and from club sports in the surrounding Philadelphia community (*Mean Age* = 21.54 (3.27) years; 62 females). Upon scanning, one participant was found to have hydrocephalus and was excluded from all subsequent behavioral and imaging analyses. The final sample of participants was predominantly White (70.37%), and not Latinx (88.89%). Additionally, our sample was recruited from 26 different sports areas spanning varying levels of engagement from college varsity, professional, club/regional, and recreational/intramural play. Of our sample, 97.22% played more than one sport, with 58.33% of the sample being in the middle of the season for their primary sport at the time of enrollment. While not all subjects were in the middle of the season for their primary sport, all athletes were current players. Moreover, this study was part of a larger research project examining the relation between concussive insult and susceptibility to substance abuse (see State of Pennsylvania, Dept. of Health CURE grant: “Mechanisms and treatment strategies to counter addiction susceptibility post TBI”).

For a summary of demographic and sport-related information see Supplementary Table 1. Prior to data collection, all methods were approved by the Institutional Review Board at Temple University. Athletes were screened to ensure they had normal or corrected-to- normal vision, and had no diagnosis of any neurological conditions, developmental delays, or disabilities.

### 2.2. Procedures

Subjects completed questionnaires, neuropsychological evaluations, and mood inventories in person at Temple University. The behavioral gamut lasted approximately 1 h during which subjects completed all computerized measures before undergoing paper-and-pencil tasks administered by a trained assessor. Either on the same day or within 1 week of behavioral testing, subjects also completed their MRI scan at the Temple University Brain Imaging Center (TUBRIC). This session lasted 1 h and included the anatomical and diffusion imaging scans used in the current analysis. More details on the behavioral and MRI evaluations are delineated below.

### 2.3. Concussion history

Concussion history was determined by responses on the OSU TBI-ID (Rosenthal et al., 2014; Corrigan and Bogner, 2018) structured interview. Concussion was operationalized as the endorsement of any head-impact resulting in dazed confusion and/or memory gaps, and/or a loss of consciousness not exceeding 30 min. On average participants reported 2 concussions (range = 1–6). A summary of the characterization of our sample on the basis of concussive and medical history is provided below (see Supplementary Table 2). On average, participants with concussion reported that their (last) concussion occurred 57 months ago (range 1–336 months post-concussion). Additionally, only seven subjects could be considered in the sub-acute stage of injury, having incurred a concussion within 1–6 months of enrollment. Due to the heterogeneity of our sample’s concussion profile, we chose to look at OSU TBI-ID metrics based on discrete groups (i.e., NCHx, SCHx, MCHx, and RHx), as well as in a continuous manner. Subjects were not excluded on the basis of remoteness or acuteness or injury, as we consider this informative variability reflective of a cohort one my find presenting at a clinic. That is, the inclusion of these subjects provides a basis for greater ecological validity.

In order to investigate the effects of concussion on mood, cognition, and brain health in an exhaustive fashion, detailed OSU TBI-ID metrics were extracted, including raw number of concussive injuries, number of losses of consciousness, time since last injury, and age at first injury. For those who experienced periods of time where repetitive, sub-threshold impacts to the head were sustained, we also calculated the age at onset and duration of the period of repetitive trauma.

### 2.4. Mood and anxiety

Mood and anxiety symptoms were measured using the Hospital Anxiety and Depression Scale (HADS; Zigmond and Snaith, 1983; Stern, 2014).

### 2.5. Cognitive tests

A broad spectrum of cognitive abilities was assessed using standardized neuropsychological tasks. These tasks included measures assessing the Miyake model of executive functioning (Miyake and Friedman, 2012), such as inhibition (flanker task), cognitive flexibility (set-shifting), and working memory (N-back) taken from the NIH EXAMINER battery (Kramer, 2014). The inhibition/Flanker total score, reflecting both accuracy and response speed for incongruent trials was computed according to procedures described in the EXAMINER User Manual. First, an accuracy score representing the proportion of correct responses (out of 24 trials), multiplied by 5 was computed to create a score ranging from 0 to 5. Reaction times for correct incongruent trials were truncated between 500 and 3,000 ms, and then log values were algebraically rescaled from a log (500) – log (3,000) range to a 0–5 range, with faster times yielding higher scores. The accuracy and response time scores were summed to create a total composite ranging from 0 to 10 points, with higher scores assigned for more accurate trials and faster response times. The same method was used to compute an accuracy and reaction time composite score for the Set Shifting task. A discriminability index (d prime) was computed to measure performance on the N-back test as the difference between the hit rate and the false positive rate. Additionally, processing speed was measured with the Symbol-Digit Modalities Task (SDMT; Smith, 1982; total correct in 90 s). Non-verbal IQ was measured with the matrices subtask from the KBIT-2 (Kaufman and Kaufman, 2004; standard score based on age-based norms for total correct), and episodic memory using the Hopkins Verbal Learning Task (HVLT; Belkonen, 2011; total correct on free recall trials).

### 2.6. Behavioral data analysis

Non-parametric correlations between the OSU TBI-ID metrics, control indices, mood, and cognition were conducted using Spearman’s rho (ρ), as this test is robust to violations of normality, making it better suited to account for outliers. For group-level comparisons Welch’s *t*-tests are reported for all neuropsychological and clinical measures, as this test is robust to violations of the homogeneity of variances assumption. Data were segregated to look at differences between those who have never sustained a concussion (NCHx), suffered one lifetime concussive insult (SCHx), and endured multiple concussions (MCHx). Furthermore, *post hoc* follow-up contrasts were performed between the aforementioned three groups and those who underwent a period of repetitive, non-concussive head impacts (RHx). Those belonging to this group may or may not have had some lifetime history of some diagnosed concussion. Note that when data were non- normally distributed, Mann–Whitney U-tests are reported.

For correlations, multiple comparisons are controlled for using the false discovery rate (FDR) correction for every time a control, cognitive, or mood variable is newly correlated with another OSU TBI-ID measure. That is, since there are six OSU TBI-ID variables under study, control, cognitive, and mood *p*-values were vectorized such that 6 values were factored into the correction for each comparison (e.g., age is correlated with 6 OSU TBI-ID measures, so every age- related *p*-value is included in the correction for that variable). This was similarly repeated for pairwise contrasts, such that FDR *p*-values were calculated by vectorizing each measure based on the number of contrasts being performed on a given variable (i.e., 3 times for the *a priori* contrasts, and 3 times for the *post hoc* contrasts, respectively).

### 2.7. Brain image acquisition

During the scan, padding was placed around participants’ heads to reduce motion. Participants were scanned in a Siemens 3.0 T scanner (MAGNETOM Trio Tim System, Siemens Medical Solutions, Erlangen, Germany) with a 64-channel phased-array parallel coil. During scans, participants watched a TV show in order to divert attention and reduce movement.

Image acquisition included a T1 magnetization-prepared rapid gradient-echo (MPRAGE) sequence (176 contiguous sagittal slices, 0.9 mm isotropic voxel size; 1900 ms TR; 2.32 ms TE; 9-degree flip angle; 256 × 256-pixel matrix). T1 images were checked immediately following the scan to ensure high data quality.

Diffusion images were acquired with a hybrid imaging sequence with a parallel imaging mode (GRAPPA) at an acceleration factor of 2. The diffusion scheme comprised of 145 non-collinear diffusion-weighted acquisitions. Of these, the volumes consisted of 6 b = 250 s/mm^2^, 21 b = 1000 s/mm^2^, 24 b = 2000 s/mm^2^, 30 b = 3250 s/mm^2^, 61 b = 5000 s/mm^2^ and 3 T2-weighted *b* = 0 s/mm^2^ acquisitions (2683 ms TR; 83.6 ms TE; 128 × 128 matrix; 69 slices with 2 mm isotropic voxels). Additionally, non-diffusion-weighted field-maps with anterior to posterior and inverse phase-encoding directions were collected to measure echo-planar imaging (EPI) distortions. These images consisted of two b0 volumes each. All other parameters for field-map acquisition were matched to that of our diffusion-weighted volumes.

### 2.8. Brain image processing

#### 2.8.1. Diffusion-weighted imaging

Diffusion-weighted images were processed using tools in the FMRIB Software Library (FSL v6.0.2; Image Analysis Group, FMRIB, Oxford, UK).^1^ Using the FMRIB Diffusion Toolbox, susceptibility artifacts, EPI distortions, subject motion and eddy current-induced distortions were corrected (Andersson et al., 2003; Andersson and Sotiropoulos, 2016). A binary brain mask was created by removing the non-brain tissue with FSL’s Brain Extraction Tool from each participant’s topup-corrected, time-collapsed b0 image. The most popular DWI metric is fractional anisotropy (FA; Nir et al., 2017), a measure of how directionally constrained or unconstrained water diffusion is in a given voxel (Murphy and Frodl, 2011). Other well-established metrics that have shown relevance for characterizing the neural etiology of concussive injury include mean diffusivity (MD) or the overall magnitude of diffusivity within a voxel (Clark et al., 2011), and radial diffusivity (RD), or the degree of diffusivity that runs perpendicular to the orientation of the underlying fibers (Winklewski et al., 2018). Eigenvector and eigenvalues along with FA, MD, and RD, were computed in native anatomical space using the dtifit program (Pierpaoli et al., 1996). Longitudinal diffusivity (λ1), or axial diffusivity (AD) was also included in this analysis and can be conceptualized as the degree of diffusion parallel to the underlying fiber tract (Winklewski et al., 2018). It is important to note that prior to fitting the tensor model, data volumes with b up to 1000 were extracted, as the tensor model tends to fall apart with higher diffusion weightings (Crombe et al., 2022).

#### 2.8.2. Free-water correction

In order to disentangle the differential contribution of extracellular free-water in the pathophysiology of concussion, a free-water correction was applied to all principal scalars. We performed the free-water correction in three steps. First, it was paramount that the data be denoised and de-Gibbsed in Mrtrix3, as failing to do so resulted in extraneous ventricular noise post- correction. Then, data volumes were extracted up to b of 2000, as diffusion weightings up to this value are critical for the imaging of the cerebral spinal fluid (CSF) tissue compartment (Pasternak et al., 2009; Hoy et al., 2014; Hoffman et al., 2022). Finally, the free-water correction was performed using Diffusion Imaging in Python (DIPY).^2^ Voxels containing a free- water volume fraction (FW) higher than 0.7 were set to zero, they had the highest probability concentration of free-water contamination, and mostly belonged to the ventricles. This step yielded all free-water-corrected principal scalars (i.e., FAt, MDt, RDt, ADt; -t stands for “tissue”), as well as a free-water volume fraction map (FW). To our knowledge, only one study uses this method in the context of acute concussive injury (scanned within 72 h of injury; Pasternak et al., 2014). This is the first retrospective study of its kind to use free-water DTI (fwDTI) in the context of retrospective concussive abnormalities. Moreover, it is important to note that this is the first dMRI investigation of concussion to look at MDt in particular (Pasternak et al., 2014).

#### 2.8.3. Tract-based spatial statistics (TBSS) image processing and statistical analysis

Whole-brain DWI data were analyzed using tract-based spatial statistics (TBSS) analysis (Smith et al., 2006). All participants’ FA images were organized and preprocessed, a procedure that eliminates potential outliers that result from the tensor-fitting process. Subsequently, all preprocessed FA maps were non-linearly registered to the 1 × 1 × 1 FMRIB58_FA image in FSL’s built in MNI repository. After registration to the aforementioned target, all FA images were aggregated into a composite 4D file, after which point all volumes in this image were averaged together to get the mean FA of the sample. The latter image was ultimately used to derive an average FA skeleton for our cohort, which was thresholded at a level of 0.2 before any voxel-wise paired-subjects *t*-tests were performed (described below).

It is important to note that only a subset of the overall sample of 107 subjects was used in the final imaging analysis, as not all participants completed the MRI session of the neuroimaging arm, and some data needed to be removed from analysis due to excessive intensity artifacts or motion. The final imaging analysis consisted of 86 participants, yielding a design matrix for voxel- wise statistics that was configured such that the control group consisted of 32 subjects who had no history of concussive injury, while the patient group consisted of 54 subjects who had sustained at least 1 concussion over the course of their lifetime. All contrasts were performed with 5,000 permutations using FSL’s *randomize* tool (Winkler et al., 2014) in FSL along with the Threshold- Free Cluster Enhancement option, in order to impose a more rigorous alternative to traditional cluster-based thresholding techniques (Smith and Nichols, 2009). Between-group voxel-wise statistics were repeated similarly for FA, MD, RD, AD, FAt, MDt, RDt, ADt, and FW. All reported *randomize* statistics have been thoroughly spatially corrected for multiple comparisons. Though *p*-values are derived through 5,000 permutations, yielding more stable test statistics, further correction was performed using FDR correction due to the sheer number of comparisons under study. The *p*-values were vectorized for each contrast within each scalar (resulting in an array of 6 values, as 6 contrasts were performed per scalar). Additionally, due to the large number of contrasts run, we summarized our results with the assistance of the *pnl_randomise* software packaged developed by Kang-Ik Kevin Cho.^3^

#### 2.8.4. Whole-brain scalars and correlations with OSU TBI-ID metrics

In order to assess the effects of the different OSU TBI-ID measures against the health of cerebral white matter, whole-brain metrics were extracted for each subject on every principal and free-water corrected scalar. These measures were generated by warping subjects’ data to a standard space FA template (i.e., FMRIB58_FA image) in FSL, and overlaying the TBSS white-matter skeleton over each scalar, averaging the scalar values in each voxel of the skeleton. It is important to note that while this skeleton contains the most prominent white matter tracts in the brain, it does not yield a true measure of all the brain’s white matter. This is due to the fact that it does not account for much of small u-shaped association fibers. Therefore, it is important to stress the resultant measures are a simply a close approximation of true whole-brain white matter. Multiple comparisons are controlled for by vectorizing the *p*-values of each whole-brain scalar for every time it is correlated with a different OSU TBI-ID measure (e.g., FA is correlated with 6 different OSU TBI-ID measures, so every FA-related *p*-value is vectorized in an array of 6 values for the correction).

## 3. Results

### 3.1. OSU TBI-ID validation

#### 3.1.1. Correlations with controls measures, tests of cognitive abilities, and mood

These results are summarized in full in Supplementary Table 3. Due to the large number of correlations, and abridged summary of our findings are summarized in narrative form below.

First, number of concussions was positively correlated with age (*rho* = 0.23, *p* = 0.018, 95% CI [0.04, 0.40]), though upon correcting for multiple comparisons this association become trending (*pcorr* = 0.054). Moreover, number of concussions was negatively associated with flanker performance, such that performance became worse with increasing number of injuries (*rho* = −0.20, *p* = 0.045, 95% CI [−0.37, −0.01]). However, this relation did not survive correction for multiple comparisons (*pcorr* = 0.270).

Performance on the third trial of the HVLT was positively associated with number of losses of consciousness amongst those who had sustained at least one concussive injury (*rho* = 0.28, *p* = 0.025, 95% CI [0.04, 0.48]; note that those who had never sustained an injury were excluded from this analysis to mitigate the pooling of values at 0). It is also important to note that there was a trending relation between performance on the delayed HVLT and number of losses of consciousness (*rho* = 0.23, *p* = 0.068, 95% CI [−0.02, 0.44]). However, none of these findings survived FDR correction.

Similarly, for time since last injury, only those who had sustained at least one concussion were included in the analysis. There were significant associations between this metric and age (*rho* = 0.42, *p* < 0.001, 95% CI [0.20, 0.60]) and HADS anxiety scores (*rho* = −0.28, *p* = 0.023, 95% CI [−0.49, −0.04]). Only the association with age survived the FDR correction (*pcorr* = 0.003), whereas the association with anxiety symptoms did not (*pcorr* = 0.138).

Additionally, for those who sustained at least one injury, there was an inverse association with number of sports played (*rho* = −0.30, *p* = 0.017, 95% CI [−0.50, −0.06]) and with the third trial of the HVLT (*rho* = −0.26, *p* = 0.035, 95% CI [−0.47, −0.02]). Neither the association between age at first injury and number of sports played (*pcorr* = 0.138), nor that between age at first injury and HVLT trial 3 (*pcorr* = 0.105) survived multiple comparisons corrections.

Finally, there were no significant correlations between the two variables derived from section three of the OSU TBI-ID concerning details surrounding incidents of repetitive, subclinical trauma. More specifically, there were no associations between age at onset of repetitive trauma, nor duration of period of repetitive trauma. These correlations were performed only in those who espoused having undergone some period of repetitive injury that did not result in dazed confusion, memory gap, or loss of consciousness.

#### 3.1.2. Correlations with whole-brain principal and free-water corrected scalars

After visual inspection of the diffusion data, 19 participants were excluded from the final analysis due to excessive motion or intensity artifacts, yielding a final N of 88. Note that for correlations where the N was below 88, the number of subjects was such due to either the exclusion of those who never sustained a concussion, or missing data for certain OSU TBI-ID measures. There were no significant correlations between TBSS-derived whole-brain or free-water corrected scalars and any measure of concussion or subclinical repetitive trauma. For a complete summary of these results and descriptives for all scalars, see Tables 1, 2, respectively.

**TABLE 1:** Correlations between OSU measures and whole-brain DWI scalars.

OSU metrics and whole brain metrics					
Variable	*N*	Spearman’s rho (ρ)	P (Pcorr)	95% CI	
				Lower	Upper
**1. Number of concussions**					
**Traditional DTI scalars**					
FA	88	−0.04	0.695 (0.943)	−0.25	0.17
MD		0.04	0.747 (0.833)	−0.18	0.24
AD		0.02	0.851 (0.950)	−0.19	0.23
RD		0.02	0.824 (0.846)	−0.19	0.23
**Free-water imaging scalars**					
FW	88	0.03	0.819 (0.926)	−0.19	0.23
FAt		−0.03	0.785 (0.902)	−0.24	0.18
MDt		0.09	0.411 (0.730)	−0.12	0.29
ADt		0.09	0.419 (0.708)	−0.12	0.29
RDt		0.11	0.331 (0.662)	−0.11	0.31
**2. Number of LOC**					
**Traditional DTI scalars**					
FA	88	0.01	0.937 (0.943)	−0.20	0.22
MD		−0.02	0.833 (0.833)	−0.23	0.19
AD		−0.01	0.950 (0.950)	−0.22	0.20
RD		−0.04	0.685 (0.846)	−0.25	0.17
**Free-water imaging scalars**					
FW	88	−0.02	0.866 (0.926)	−0.23	0.19
FAt		0.03	0.791 (0.902)	−0.18	0.24
MDt		−0.04	0.721 (0.730)	−0.25	0.17
ADt		−0.03	0.802 (0.802)	−0.24	0.18
RDt		−0.04	0.695 (0.834)	−0.25	0.17
**3. Time since injury**					
**Traditional DTI scalars**					
FA	54	0.05	0.731 (0.943)	−0.22	0.31
MD		−0.05	0.703 (0.833)	−0.32	0.22
AD		−0.09	0.532 (0.950)	−0.35	0.19
RD		−0.03	0.846 (0.846)	−0.29	0.24
**Free-water imaging scalars**					
FW	54	−0.02	0.881 (0.926)	−0.29	0.25
FAt		0.06	0.665 (0.902)	−0.21	0.32
MDt		−0.05	0.730 (0.730)	−0.31	0.22
ADt		−0.04	0.796 (0.802)	−0.30	0.23
RDt		−0.02	0.870	−0.29	0.25
**4. Age at first injury**					
**Traditional DTI scalars**					
FA	54	0.13	0.358 (0.943)	−0.15	0.38
MD		−0.15	0.278 (0.833)	−0.40	0.12
AD		−0.09	0.524 (0.950)	−0.35	0.18
RD		−0.18	0.205 (0.846)	−0.42	0.10
**Free-water imaging scalars**					
FW	54	−0.12	0.388 (0.926)	−0.38	0.15
FAt		0.06	0.650 (0.902)	−0.21	0.33
MDt		−0.19	0.173 (0.730)	−0.43	0.08
ADt		−0.18	0.191 (0.660)	−0.43	0.09
RDt		−0.22	0.117 (0.662)	−0.46	0.06
**5. Age at onset of repetitive trauma**					
**Traditional DTI scalars**					
FA	19	−0.02	0.943 (0.943)	−0.47	0.44
MD		0.10	0.689 (0.833)	−0.37	0.53
AD		−0.02	0.941 (0.950)	−0.47	0.44
RD		0.14	0.563 (0.846)	−0.33	0.56
**Free-water imaging scalars**					
FW	19	0.02	0.926 (0.926)	−0.44	0.47
FAt		−0.18	0.458 (0.902)	−0.59	0.30
MDt		−0.13	0.586 (0.730)	−0.55	0.34
ADt		−0.18	0.472 (0.708)	−0.58	0.30
RDt		−0.10	0.685 (0.834)	−0.53	0.37
**6. Duration of period of repetitive trauma**					
**Traditional DTI scalars**					
FA	18	−0.18	0.465 (0.943)	−0.60	0.31
MD		0.13	0.615 (0.833)	−0.36	0.56
AD		0.24	0.340 (0.950)	−0.26	0.64
RD		0.11	0.660 (0.846)	−0.38	0.55
**Free-water imaging scalars**					
FW	18	0.19	0.450 (0.926)	−0.30	0.60
FAt		−0.03	0.902 (0.902)	−0.49	0.44
MDt		0.24	0.333 (0.730)	−0.25	0.64
ADt		0.30	0.220 (0.660)	−0.19	0.68
RDt		0.24	0.328 (0.662)	−0.25	0.64

**TABLE 2:** Below are whole-brain descriptive statistics for each group under study.

Scalar group comparison by mean								
Measure	Mean				SE			
	NCHx *n* = 33	SCHx *n* = 22	MCHx *n* = 33	RHx *n* = 16	NCHx	SCHx	MCHx	RHx
**Traditional DTI scalars**								
FA	0.389	0.389	0.389	0.391	0.002	0.002	0.002	0.002
MD	7.888E−4	7.864E−4	7.892E−4	7.853E−4	2.201E−6	3.077E−6	2.896E−6	4.700E−6
AD	1.138E−3	1.136E−3	1.139E−3	1.136E−3	2.451E−6	3.902E−6	2.932E−6	4.636E−6
RD	6.142E−4	6.117E−4	6.145E−4	6.099E−4	2.325E−6	3.319E−6	3.078E−6	4.995E−6
**Free-water imaging scalars**								
FW	0.236	0.238	0.237	0.236	0.001	0.003	0.002	0.004
FAt	0.465	0.465	0.465	0.466	0.002	0.002	0.002	0.002
MDt	6.923E−4	6.421E−4	7.143E−4	6.995E−4	1.097E−5	1.364E−5	1.660E−5	2.304E−5
ADt	1.227E−3	1.108E−3	1.265E−3	1.222E−3	2.725E−5	3.075E−5	3.730E−5	4.970E−5
RDt	4.255E−4	4.090E−4	4.393E−4	4.383E−4	4.271E−6	5.465E−6	7.692E−6	1.087E−5

Note that the RHx group is a subset of the overall sample and contains both those who have and have not sustained at least one concussion over the course of their lifetime.

### 3.2. Primary analyses: Group comparisons between NCHx, SCHx, and MCHx

#### 3.2.1. Focused contrasts on control measures, tests of cognitive abilities, and mood

In comparing NCHx and SCHx, there were trending differences in subjects’ performance on HVLT trials two (*t*(46.55) = 1.91, *p* = 0.062, *d* = 0.41, 95% CI [−0.01, 0.97]) and three (*t*(48.38) = 1.95, *p* = 0.057, *d* = 0.49, 95% CI [−0.002, 0.97]), such that those in the NCHx group performed better on tests of memory. However, none of this survived the FDR correction.

Moreover, in comparing the NCHx and MCHx groups, there was a significant difference between groups on the basis of age (*U* = 533.50, *p* = 0.022, *r*_*rb*_= −0.30, 95% CI [−0.52, −0.05]), such that the MCHx was older. Upon correcting for multiple comparisons, this effect became trending (*pcorr* = 0.066). Additionally, there was a trending difference between groups on the flanker (*U* = 926.50, *p* = 0.058, *r*_*rb*_ = −0.25, 95% CI −0.002, 0.48]), such that the MCHx group performed worse than the NCHx group. There was also a trending difference between groups on the set-shifting task (*t*(71.58) = 1.80, *p* = 0.076, *d* = 0.41, 95% CI [−0.04, 0.86]) such that the MCHx group performed worse than the NCHx group. However, none of this survived FDR correction.

Finally, there was a trending difference between the SCHx and MCHx group on the basis of age (*U* = 404.50, *p* = 0.062, *r_*rb*_* = −0.27, 95% CI [−0.50, 0.01]) that remained trending after FDR correction (*pcorr* = 0.093). Given this trend, those in the MCHx group were older than those in the SCHx group. There was also a trending difference between groups on the basis of number of sports played (*U* = 393.50, *p* = 0.062, *r_*rb*_* = −0.51, 95% CI [−0.51, 0.01]), such that those in the MCHx group played more sports than those in the SCHx group. However, this trend did not survive FDR correction. For a complete summary of results for these contrasts and descriptive statistics for each measure, see Tables 3, 4, respectively.

**TABLE 3:** Summary of pairwise comparison statistics for all metrics under study.

Pairwise comparisons by group										
Measure	*N*	DF	*t*	*U*	Mean diff.	SE	*P (Pcorr)*	*d/r_*rb*_*	95% CI	
									Lower	Upper
**NCHx vs. SCHx**										
**Control variables**										
Age	69	–	–	562.00	–	–	0.829 (0.829)	−*0.03*	−*0.30*	*0.24*
KBIT (IQ)	68	57.50	1.55	–	4.96	3.21	0.127 (0.381)	0.38	−0.11	0.87
Number of sports	69	–	–	655.50	–	–	0.356 (0.356)	*0.13*	−*0.15*	*0.39*
**Cognitive variables**										
**NIH examiner battery**										
Flanker	69	–	–	699.50	–	–	0.148 (0.222)	−*0.21*	−*0.07*	*0.45*
2-Back		57.40	0.54	–	0.08	0.14	0.589 (0.589)	0.13	−0.35	0.61
Set-shifting	68	60.86	1.63	–	0.27	0.17	0.109 (0.164)	0.40	−0.09	0.89
**Processing speed**										
SDMT	68	47.65	1.41	–	3.56	2.52	0.165 (0.248)	0.36	−0.13	0.84
**Hopkins verbal learning test**										
Trial 1	69	66.41	1.28	–	0.46	0.36	0.205 (0.491)	0.31	−0.18	0.79
Trial 2		46.55	1.91	–	0.78	0.41	0.062. (0.186)	0.41	−0.01	0.97
Trial 3		48.38	1.95	–	0.67	0.34	0.057. (0.166)	0.49	−0.002	0.97
Delayed recall		56.95	0.81	–	0.40	0.49	0.422 (0.465)	0.20	−0.28	0.68
**Psychological variables**										
**Hospital anxiety and depression scale**										
Anxiety	69	56.69	−1.44	–	−1.45	1.00	0.154 (0.231)	−0.36	−0.84	0.13
Depression		56.88	−0.91	–	−0.55	0.61	0.368 (0.683)	−0.22	−0.70	0.26
**NCHx vs. MCHx**										
**Control variables**										
Age	78	–	–	533.50	–	–	0.022* (0.066.)	−*0.30*	−*0.51*	−*0.05*
KBIT (IQ)		73.34	1.10	–	3.47	3.15	0.273 (0.410)	0.25	−0.20	0.70
Number of sports	77	–	–	646.00	–	–	0.335 (0.356)	−*0.13*	−*0.37*	*0.13*
**Cognitive variables**										
**NIH examiner battery**										
Flanker	77	–	–	926.50	–	–	0.058. (0.174)	−*0.25*	−*0.002*	*0.48*
2-Back	76	72.28	0.99	–	0.17	0.17	0.326 (0.589)	0.23	−0.23	0.68
Set-shifting	78	71.58	1.80	–	0.25	0.14	0.076. (0.164)	0.41	−0.04	0.86
**Processing speed**										
SDMT	77	73.25	−0.29	–	−0.59	2.00	0.770 (0.770)	−0.07	−0.51	0.38
**Hopkins verbal learning test**										
Trial 1	78	75.85	0.99	–	0.37	0.37	0.327 (0.491)	0.22	−0.22	0.67
Trial 2		70.40	0.79	–	0.27	0.34	0.433 (0.433)	0.18	−0.27	0.62
Trial 3		73.53	0.31	–	0.09	0.28	0.758 (0.758)	0.07	−0.37	0.51
Delayed recall		73.99	−0.73	–	−0.29	0.39	0.465 (0.465)	−0.17	−0.61	0.28
**Psychological variables**										
**Hospital anxiety and depression scale**										
Anxiety	78	75.92	−1.44	–	−1.25	0.87	0.154 (0.231)	−0.33	−0.77	0.12
Depression		75.98	−0.58	–	−0.30	0.52	0.564 (0.683)	−0.13	−0.58	0.31
**SCHx vs. MCHx**										
**Control variables**										
Age	67	–	–	404.50	–	–	0.062. (0.093.)	−*0.27*	−*0.50*	*0.01*
KBIT (IQ)	66	61.73	−0.43	–	−1.49	3.45	0.667 (0.667)	−0.11	−0.60	0.38
Number of sports		–	–	393.50	–	–	0.062. (0.186)	−*0.51*	−*0.51*	*0.01*
**Cognitive variables**										
**NIH examiner battery**										
Flanker	66	–	–	574.50	–	–	0.628 (0.628)	*0.07*	−*0.21*	*0.34*
2-Back	65	60.91	−0.61	–	−0.11	0.18	0.544 (0.589)	−0.15	−0.65	0.34
Set-shifting	67	63.42	1.12	–	0.17	0.15	0.267 (0.267)	0.27	−0.21	0.76
**Processing speed**										
SDMT	67	52.57	−1.58	–	−4.14	2.63	0.121 (0.248)	−0.39	−0.88	0.10
**Hopkins verbal learning test**										
Trial 1	67	64.84	−0.25	–	−0.09	0.36	0.806 (0.806)	−0.06	−0.54	0.42
Trial 2		55.65	−1.16	–	−0.51	0.44	0.251 (0.376)	−0.29	−0.77	0.20
Trial 3		53.82	−1.62	–	−0.58	0.36	0.111 (0.166)	−0.41	−0.89	0.09
Delayed recall		49.27	−1.48	–	−0.69	0.46	0.144 (0.432)	−0.37	−0.86	0.12
**Psychological variables**										
**Hospital anxiety and depression scale**										
Anxiety	67	56.32	0.20	–	0.20	1.01	0.845 (0.845)	0.05	−0.44	0.53
Depression		55.94	0.41	–	0.25	0.61	0.683 (0.683)	0.10	−0.38	0.59

Significance flag: *p* < 0.10, *p < 0.05, **p < 0.01, ***p < 0.001. Age and the flanker are the only variables for which a Mann–Whitney *U* test is reported due to non-normality of the data. For these two analyses, note that *rank-biserial correlation* (*r*_*rb*_) is reported (as denoted by italics) as a measure of effect size – not Cohen’s d, as is the case for all other comparisons.

**TABLE 4:** Summary of all descriptive statistics for each pairwise comparison.

Group descriptives on cognition and mood								
Measure	Mean				SE			
	NCHx *n* = 40	SCHx *n* = 29	MCHx *n* = 38	RHx *n* = 22	NCHx	SCHx	MCHx	RHx
**Control variables**								
Age	20.80	21.10	22.66	21.96	0.38	0.59	0.63	0.71
KBIT (IQ)	110.00	105.04	106.53	107.46	2.04	2.48	2.40	3.46
Number of sports	4.95	4.55	5.76	5.00	0.36	0.42	0.56	0.54
**Cognitive variables**								
**NIH^f^ examiner battery**								
Flanker	9.14	8.90	8.92	8.82	0.05	0.14	0.09	0.09
2-Back	1.75	1.47	1.58	1.58	0.11	0.13	0.12	0.14
Set-shifting	9.03	8.95	8.78	8.87	0.09	0.11	0.11	0.12
**Processing speed**								
SDMT^g^	61.28	57.72	61.87	62.36	1.31	2.16	1.51	1.60
**Hopkins verbal learning test**								
Trial 1	7.52	7.07	7.16	7.32	0.26	0.24	0.27	0.36
Trial 2	9.95	9.17	9.68	9.64	0. 21	0.35	0.27	0.40
Trial 3	10.88	10.21	10.79	10.46	0.18	0.29	0.21	0.35
Delay recall	9.95	9.55	10.24	9.86	0.30	0.39	0.25	0.40
**Psychology variables**								
**Hospital anxiety and depression scale**								
Anxiety	6.28	7.72	7.53	8.82	0.61	0.80	0.62	0.96
Depression	2.28	2.83	2.58	2.82	0.37	0.48	0.37	0.55

For the two tests that required a non-parametric assessment of group differences, the mean rank for each group is reported. Standard error in all cases refers to the standard error of the mean. Note that changes in the *n* for each group reflect either missing data or outlier removal.

#### 3.2.2. Focused TBSS contrasts on principal and free-water correct scalars

Given that only 88 subjects yielded usable dMRI data, the final design matrix consisted of 33 subjects in the NCHx group, 22 subjects in the SCHx group, and 33 subjects in the MCHx group. TBSS contrast results are summarized in Table 5, and are dichotomized between results for traditional DTI scalars and free-water corrected metrics. Six contrasts are reported per comparison, as TBSS *t*-tests are reported as one tail. The tail in which each comparison is conducted are denoted in columns one and two of Table 5. General linear models (GLMs) conducted in each scalar between NCHx and SCHx, NCHx and MCHx, and SCHx and MCHx did not reveal any significant clusters of significant difference across the entire white matter skeleton generate by the imaging software. Note that in Table 5, the maximum contrast value is noted in the fourth column, as this is the value that is used to compute *p* by subtracting it from 1, which is listed in the fifth and final column.

**TABLE 5:** Below is a summary of the TBSS contrasts for the main study groups.

TBSS contrast results				
Traditional DTI scalars				
Contrast	Comparison	Modality	Significance max	*P*-value: 1 – Max (Pcorr)
1, −1, 0	NCHx > SCHx	FA	0.385	0.615 (0.738)
−1, 1, 0	NCHx < SCHx		0.462	0.538 (0.738)
1, 0, −1	NCHx > MCHx		0.691	0.309 (0.738)
−1, 0, 1	NCHx < MCHx		0.667	0.333 (0.738)
0, 1, −1	SCHx > MCHx		0.608	0.392 (0.738)
0, −1, 1	SCHx < MCHx		0.244	0.756 (0.756)
1, −1, 0	NCHx > SCHx	MD	0.381	0.619 (0.619)
−1, 1, 0	NCHx < SCHx		0.507	0.493 (0.619)
1, 0, −1	NCHx > MCHx		0.471	0.529 (0.619)
−1, 0, 1	NCHx < MCHx		0.765	0.235 (0.619)
0, 1, −1	SCHx > MCHx		0.647	0.353 (0.619)
0, −1, 1	SCHx < MCHx		0.662	0.338 (0.619)
1, −1, 0	NCHx > SCHx	RD	0.436	0.564 (0.564)
−1, 1, 0	NCHx < SCHx		0.466	0.534 (0.564)
1, 0, −1	NCHx > MCHx		0.450	0.550 (0.564)
−1, 0, 1	NCHx < MCHx		0.787	0.213 (0.564)
0, 1, −1	SCHx > MCHx		0.501	0.499 (0.564)
0, −1, 1	SCHx < MCHx		0.666	0.334 (0.564)
1, −1, 0	NCHx > SCHx	AD	0.452	0.548 (0.548)
−1, 1, 0	NCHx < SCHx		0.621	0.379 (0.548)
1, 0, −1	NCHx > MCHx		0.501	0.499 (0.548)
−1, 0, 1	NCHx < MCHx		0.526	0.474 (0.548)
0, 1, −1	SCHx > MCHx		0.839	0.161 (0.548)
0, −1, 1	SCHx < MCHx		0.607	0.393 (0.548)
**Free-water imaging scalars**				
1, −1, 0	NCHx > SCHx	FW	0.133	0.867 (0.867)
−1, 1, 0	NCHx < SCHx		0.785	0.215 (0.430)
1, 0, −1	NCHx > MCHx		0.309	0.691 (0.829)
−1, 0, 1	NCHx < MCHx		0.807	0.193 (0.430)
0, 1, −1	SCHx > MCHx		0.786	0.214 (0.430)
0, −1, 1	SCHx < MCHx		0.614	0.386 (0.579)
1, −1, 0	NCHx > SCHx	FAt	0.375	0.625 (0.804)
−1, 1, 0	NCHx < SCHx		0.601	0.399 (0.798)
1, 0, −1	NCHx > MCHx		0.640	0.360 (0.798)
−1, 0, 1	NCHx < MCHx		0.330	0.670 (0.804)
0, 1, −1	SCHx > MCHx		0.780	0.220 (0.798)
0, −1, 1	SCHx < MCHx		0.186	0.814 (0.814)
1, −1, 0	NCHx > SCHx	MDt	0.759	0.241 (0.798)
−1, 1, 0	NCHx < SCHx		0.202	0.798 (0.798)
1, 0, −1	NCHx > MCHx		0.559	0.441 (0.798)
−1, 0, 1	NCHx < MCHx		0.381	0.619 (0.798)
0, 1, −1	SCHx > MCHx		0.299	0.701 (0.798)
0, −1, 1	SCHx < MCHx		0.706	0.294 (0.798)
1, −1, 0	NCHx > SCHx	RDt	0.769	0.231 (0.693)
−1, 1, 0	NCHx < SCHx		0.345	0.655 (0.874)
1, 0, −1	NCHx > MCHx		0.272	0.728 (0.874)
−1, 0, 1	NCHx < MCHx		0.633	0.367 (0.734)
0, 1, −1	SCHx > MCHx		0.106	0.894 (0.894)
0, −1, 1	SCHx < MCHx		0.868	0.132 (0.693)
1, −1, 0	NCHx > SCHx	ADt	0.705	0.295 (0.660)
−1, 1, 0	NCHx < SCHx		0.152	0.848 (0.848)
1, 0, −1	NCHx > MCHx		0.603	0.397 (0.660)
−1, 0, 1	NCHx < MCHx		0.500	0.500 (0.660)
0, 1, −1	SCHx > MCHx		0.450	0.550 (0.660)
0, −1, 1	SCHx < MCHx		0.555	0.445 (0.660)

### 3.3. Secondary analyses: Group comparisons between three main groups and RHx

#### 3.3.1. Focused contrasts on control measures, tests of cognitive abilities, and mood

First, there was a significant difference between the ages of the NCHx and RHx groups, such that the NCHx group was younger (*U* = 214.50, *p* = 0.017, *r_*rb*_* = −0.39, 95% CI [−0.63, −0.09]), which became trending after the FDR correction (*pcorr* = 0.051). Moreover, there was a significant difference between these groups based on flanker performance such that the RHx group performed worse (*U* = 472.00, *p* = 0.013, *r_*rb*_* = 0.42, 95% CI [0.12, 0.66]), and anxiety scores such that the RHx group had higher anxiety symptoms (*t*(28.55) = −2.29, *p* = 0.030, *d* = −0.67, 95% CI [−1.25, −0.08]). Both of these effects survived multiple comparisons corrections (flanker *pcorr* = 0.039; anxiety *pcorr* = 0.045).

Second, in comparing the SCHx group with the RHx group, there was a trending difference on the SDMT such that the RHx performed worse (*t*(38.50) = −1.84, *p* = 0.074, *d* = −0.55, 95% CI [−1.17, 0.07]), though this trend did not survive the FDR correction (*pcorr* = 0.222).

Finally, there were no differences on any measure between the RHx group and the MCHx group. For a complete summary of these contrasts, see Supplementary Table 4.

#### 3.3.2. Focused TBSS contrasts on principal and free-water correct scalars

Tract-based spatial statistics results between each of the three main groups (i.e., NCHx, SCHx, and MCHx) and those who suffered a period of repetitive, subclinical head impacts (RHx) is summarized in Supplementary Table 5. It is important to note that the reclassification of subjects into the RHx group yielded a design matrix where the *n* for NCHx was 32, SCHx was 17, MCHx was 23, and RHx was 16. With these groups, all group-level voxel-wise comparisons for each scalar were null in each tail (see Supplementary Table 5).

## 4. General discussion

The goal of this study was to dispassionately assess whether a history of concussion or subthreshold repetitive head trauma in a naturalistic cohort of young adult athletes was associated with any persistent deficits in cognition or white matter. Theoretical considerations helped guide our choice of behavioral metrics while also considering the reliability of the particular measures. To analyze brain white matter, we collected DWI data and analyzed it with technique called TBSS, which is a one of the most common ways to analyze diffusion data. This renders our analytical pipeline transparent and replicable. Finally, the current report has a large sample size, with a large number of female participants. This study is one of first of its kind to use the OSU TBI-ID method to probe for concussion history.

### 4.1. Control variable neuropsychological profiles of concussion and repetitive head trauma

First, out results show that those with more concussions tend to be older compared to those who never sustained a concussion, as well as those with a single concussion. This makes sense since those who are older have had more opportunity to incur an injury. In line with these results, those who are older tend to be further out from the date of their last injury, potentially reflecting that the level of engagement in sporting activities decreases with age in adulthood.

Additionally, our findings show that those with a history of repetitive subclinical impacts to the head over a relevant period of time have relatively worse outcomes on a single task: the flanker – a measure of inhibitory executive control. Moreover, the RHx group had higher levels of anxiety compared to those who never sustained a concussion. Most published research relevant to concussion in young adult athletes during the acute to sub-acute phase have focused on the effects of repeated, subclinical insults, rather than concussion *per se*, to the head. These studies report the presence of a range of behavioral deficits in the studied population: poorer memory (Lipton et al., 2013; McAllister et al., 2014), oculomotor impairments (Clough et al., 2018), and higher levels of anxiety and depression (Meier et al., 2016b; Wu et al., 2020). The literature on concussion in young adult athletes during the *chronic* phase is much smaller and quite mixed. Several studies have reported no differences in cognition (List et al., 2015; Meier et al., 2016a; Churchill et al., 2019), which is broadly consistent with our findings since most aspects of cognition were completely normal in our sample. To our knowledge only one study reported robust evidence for persistent cognitive deficits on a range of tasks including delayed memory in post-concussion former athletes (Tremblay et al., 2014). In light of this, our findings are in line with what the majority of studies in the literature suggests: that one or a few clinically significant concussions does not lead to negative long-term effects on cognition. Of course, we cannot make conjectures as to how a history of concussions may manifest downstream consequences in the far future, such as in the context of aging. The cross-sectional nature of our study also introduces a directionality problem: is it because those who find themselves in the RHx group are more prone to anxiety or issues of impulse control that these findings emerged? Or is this truly a consequence of repetitive subthreshold head trauma? Further research assessing these capacities over time are needed to better elucidate these findings.

What accounts for the differences across studies in the literature? One of the most salient sources of noise is the heterogeneous operationalizations of concussion status. For example, while many studies in the acute to sub-acute literature rely on field side diagnosis by a physician for determining concussion status, many of these reports do not address in any detail what criteria were used in the diagnostic process, particularly in the case of the Concussion Assessment, Research, and Education Consortium Project studies (Mustafi et al., 2018; Brett et al., 2019; Wu et al., 2020). Other reports have been similarly vague, citing use of symptom evaluation, along with the implementation of a seemingly custom series of assessments including a cranial nerve check, muscle strength evaluation, Rhomberg’s balance test, UPMC Center for Sports Medicine cognitive testing, and the King–Devick test (Meier et al., 2016b).

We chose to use the OSU TBI-ID to operationalize lifetime exposure to concussion. This is a standardized, open-source assessment tool that was developed based on the TBI monitoring guidelines established by the Center for Disease Control and Prevention (Gerberding and Binder, 2003). The utility of the OSU TBI-ID is further bolstered by its reliability and predictive validity (Corrigan and Bogner, 2007; Bogner and Corrigan, 2009). In light of this, and in addition to the fact that there are online training modules for the administration and interpretation of this structured interview, this tool presents a tremendous opportunity for greater consistency and objectivity in studies of sports related concussion in the retrospective stage of injury.

### 4.2. Structural connectivity and sports-related concussion and repetitive head trauma

Contrary to our expectation, our results showed that cerebral white matter in young athletes with zero, single, and multiple history of concussion were quantitatively identical to each other. Additionally, these three groups were identical to those who sustained periods of subthreshold, repetitive impacts to the head. As noted in the introduction, there is a complete lack of consensus in the diffusion imaging literature. The lack of consensus is found in studies focused on the acute phase and chronic phase. Null results like ours have been reported by other labs many times over but they tend to get ignored.

If we zoom and look at broad trends in the literature, there are three patterns. First, MD may be the best biomarker for identifying injury insofar as it is the most consistently reported as significant (note that we did not see any changes in MD). Second, changes in white matter are more commonly observed at the acute to sub-acute stage. Third, many subclinical impacts tend to lead to worse outcomes on white matter than those begotten by one (or a few) clinically significant concussive events.

It is also important to note that no voxel-wise clusters of difference were identified between our groups on any metric due to the fact that there was no way to control for where impacts to the head occurred. Due to the spatial heterogeneity of the forces that may result in a concussion, or those that simply constitute a subthreshold blow to the head, in addition to our inability to identify these factors retrospectively, our lack of findings in this domain do not necessarily indicate that brain health in our sample is pristine. Instead, these null results may reflect excessive variability in terms of the point of impact, washing out any possibility for there to be an effect. For example, one athlete might suffer a concussion by falling off a balance beam and hitting the back of their head while another athlete might be tackled from the side, causing a side-to-side profile of damage. In both instances there is a sports-related concussion, however, the neural damage would be vastly different. The most common analytical method (which we use in the current report), TBSS, requires the averaging across individuals in order to have sufficient power. Grouping individuals with different types of injuries blurs one’s ability to see fine-grained white matter damage. This would be true of every single study described in our literature review. Furthermore, it is important to note that this same heterogeneity may have resulted in our inability to find whole-brain correlations on principle and free-water corrected scalars, as both group-level and continuous investigations of head injury status yielded null microstructural results. As such, the best path forward is to take a single-subject approach using an ultra-high-angular-resolution DWI approach such as diffusion spectral imaging (DSI).

We caution readers from extending our findings on mild TBI at the chronic phase, to the acute to sub-acute stage or to moderate to severe TBI at any stage. Elapsed time as well as severity of injury are potent variables that should not be blurred over yet unfortunately are often confused in this literature.

### 4.3. Limitations

Our study was limited by three aspects of our sample population. First, having more subjects in the acute to sub-acute stage of injury would have given us the power necessary to make cross- sectional comparisons between the cognitive and physiological effects of acute (within 72 h of injury), sub-acute (within 6 months of injury) versus chronic (over 6 months since injury) consequences of concussion. Second, we found it difficult to recruit individuals who had sustained three or more concussions, resulting in a cohort consisting of mostly MCHx athletes who had mostly sustained 2 concussions. While our pair-wise comparisons either trichotomized (*a priori*) or quadrotomized (*post hoc*) the concussion variable to reflect the presence or absence and degree of injury, the recruitment of a sample with more head injuries would have potentially unveiled significant effects of multiple concussions on both the behavioral and neural metrics. Third, these results are only generalizable to healthy young adult athletes. That is, we do not aim to make claims about the effects of concussion in the context of aging, or outside the realm of SRC. It is for this reason, we do not delve into the literature on concussions sustained outside the scope of athletics (e.g., especially in military or combat contexts), or in elderly populations.

Additionally, relatively few subjects in our sample fell into the repetitive head trauma group. Because of this, we may not have had the power to detect as association between duration of repetitive head trauma, age at onset of repetitive head trauma, and cognitive outcomes of interest. Additionally, we did not have the power to detect differential outcomes between those who experienced multiple periods of time where they endured repetitive impacts to the head. Moreover, it is important to note that while the heterogeneity of the types of sports played in our cohort lends to a great deal of ecological validity, it may have introduced bias, since different types of sports engagement may beget distinct concussion-related changes. Moreover, since the majority of subjects in our cohort had at least one concussion over the course of their lifetime, the effect of concussion on white matter is difficult to tease out. Future retrospective studies of this kind should therefore aim for a larger control group.

Finally, it is important to note that our null results may have been due to the insensitivity of the neuropsychological evaluations and imaging modality under study. Moreover, while the current report delves deeply into the existent diffusion imaging literature on chronic concussion, we do not investigate the potentially utility of other imaging modalities, which have shown some promise in probing for the long-term cognitive and neurological impact of concussive injury (see Poltavski and Biberdorf, 2014; Poltavski et al., 2017, 2019). Therefore, the current results should be interpreted with caution, as they do not necessarily generalize to other imaging modalities or behavioralassessments.

## Data availability statement

The raw data supporting the conclusions of this article will be made available by the authors, without undue reservation. However, due to privacy reasons, the data will be de-identified. Further inquiries can be directed to the corresponding author.

## Ethics statement

The studies involving human participants were reviewed and approved by the Temple University Institutional Review Board (IRB). The patients/participants provided their written informed consent to participate in this study.

## Author contributions

RM and CB organized the database. RM, CB, and LH were involved in data collection. LH performed the statistical analysis, assisted in the organization of the imaging data, and wrote the first draft of the manuscript. IO and TG wrote the sections of the manuscript. All authors contributed to the conception and design of the study, assisted with manuscript revision, read, and approved the submitted version.

## Footnotes

1
https://www.fmrib.ox.ac.uk/fsl/

2
https://dipy.org

3
https://github.com/pnlbwh/pnl_randomise
