# Supplementary material for: Concussions in young adult athletes: No effect on cerebral white matter
Source: Front Hum Neurosci. 2023 Mar 1;17:1113971. doi: 10.3389/fnhum.2023.1113971 (PMC10014705; doi:10.3389/fnhum.2023.1113971)
Supplement: Supplementary file 1 [file Data_Sheet_1.DOCX]

**Supplementary Materials**

**Table S1.** Participant demographic data as well as information about sports played, and concussion characterization. Note that for the field titled “Currently Play (Is Sport in Season)?”, the values are indicating the count and percentage of people whose sport is in the middle of a season necessitating regular play. With this in mind, it is important to note that all athletes are current athletes, regardless of whether or not their season was active at the time of enrollment.

| **Demographics** | | | | | | | | | | |
| --- | --- | --- | --- | --- | --- | --- | --- | --- | --- | --- |
|  | Total (*n* = 107) | | NCHx^[[1]](#footnote-1)^a (*n* = 40) | | SCHx^[[2]](#footnote-2)^b (*n* = 29) | | MCHx^[[3]](#footnote-3)^c (*n* = 38) | | RHx^[[4]](#footnote-4)^d (*n* = 22) | |
|  | Count | Percent | Count | Percent | Count | Percent | Count | Percent | Count | Percent |
| Race | | | | | | | | | | |
| *Native American/Alaskan* | 1 | 0.93% | 0 | 0.00% | 1 | 3.45% | 0 | 0.00% | 0 | 0.00% |
| *Asian* | 7 | 6.54% | 2 | 5.00% | 1 | 3.45% | 4 | 10.53% | 0 | 0.00% |
| *Black or African American* | 14 | 13.08% | 5 | 12.50% | 6 | 20.69% | 3 | 7.89% | 3 | 13.64% |
| *Hawaiian or Pacific Islander* | 1 | 0.93% | 0 | 0.00% | 1 | 3.45% | 0 | 0.00% | 0 | 0.00% |
| *White or Caucasian* | 75 | 70.09% | 31 | 77.50% | 16 | 55.17% | 28 | 73.68% | 17 | 77.27% |
| *Unknown* | 2 | 1.87% | 0 | 0.00% | 1 | 3.45% | 1 | 2.63% | 0 | 0.00% |
| *Mixed* | 7 | 6.54% | 2 | 5.00% | 3 | 10.34% | 2 | 5.26% | 2 | 9.09% |
| Ethnicity | | | | | | | | | | |
| *Not Latinx* | 96 | 89.72% | 36 | 90.00% | 24 | 82.76% | 36 | 94.74% | 21 | 95.45% |
| *Latinx* | 7 | 6.54% | 3 | 7.50% | 3 | 10.34% | 1 | 2.63% | 0 | 0.00% |
| *Unknown or Not Reported* | 4 | 3.74% | 1 | 2.50% | 2 | 6.90% | 1 | 2.63% | 1 | 4.55% |
| Sex | | | | | | |  |  |  |  |
| *Female* | 45 | 42.06% | 15 | 37.50% | 12 | 41.38% | 18 | 47.37% | 10 | 45.45% |
| *Male* | 62 | 57.94% | 25 | 62.50% | 17 | 58.62% | 20 | 52.63% | 12 | 54.55% |
| **Sport Information** | | | | | | | | | | |
|  | Total | | NCHx | | SCHx | | MCHx | | RHx | |
|  | Count | Percent | Count | Percent | Count | Percent | Count | Percent | Count | Percent |
| Primary Sport | | | | | | | | | | |
| *Soccer* | 16 | 14.95% | 7 | 17.50% | 4 | 13.79% | 5 | 13.16% | 4 | 18.18% |
| *Track/Field* | 10 | 9.35% | 5 | 12.50% | 4 | 13.79% | 1 | 2.63% | 1 | 4.55% |
| *Lacrosse* | 7 | 6.54% | 1 | 2.50% | 2 | 6.90% | 4 | 10.53% | 3 | 13.64% |
| *Cross Country Running* | 4 | 3.74% | 3 | 7.50% | 0 | 0.00% | 1 | 2.63% | 0 | 0.00% |
| *Gymnastics* | 6 | 5.61% | 2 | 5.00% | 1 | 3.45% | 3 | 7.89% | 0 | 0.00% |
| *American Football* | 6 | 5.61% | 2 | 5.00% | 2 | 6.90% | 2 | 5.26% | 2 | 9.09% |
| *Volleyball* | 12 | 11.21% | 3 | 7.50% | 3 | 10.34% | 6 | 15.79% | 1 | 4.55% |
| *Fencing* | 1 | 0.93% | 0 | 0.00% | 1 | 3.45% | 0 | 0.00% | 0 | 0.00% |
| *Baseball* | 3 | 2.80% | 0 | 0.00% | 3 | 10.34% | 0 | 0.00% | 0 | 0.00% |
| *Rowing/Crew* | 6 | 5.61% | 3 | 7.50% | 2 | 6.90% | 1 | 2.63% | 1 | 4.55% |
| *Cheerleading* | 3 | 2.80% | 2 | 5.00% | 1 | 3.45% | 0 | 0.00% | 2 | 9.09% |
| *Ice-skating* | 1 | 0.93% | 1 | 2.50% | 0 | 0.00% | 0 | 0.00% | 0 | 0.00% |
| *Basketball* | 2 | 1.87% | 1 | 2.50% | 1 | 3.45% | 0 | 0.00% | 1 | 4.55% |
| *Tennis* | 2 | 1.87% | 1 | 2.50% | 1 | 3.45% | 0 | 0.00% | 0 | 0.00% |
| *Rugby* | 9 | 8.41% | 3 | 7.50% | 2 | 6.90% | 4 | 10.53% | 3 | 13.64% |
| *Field Hockey* | 1 | 0.93% | 0 | 0.00% | 0 | 0.00% | 1 | 2.63% | 0 | 0.00% |
| *Other Events/Activities* | 1 | 0.93% | 1 | 2.50% | 0 | 0.00% | 0 | 0.00% | 0 | 0.00% |
| *Ultimate Frisbee* | 5 | 4.67% | 1 | 2.50% | 1 | 3.45% | 3 | 7.89% | 0 | 0.00% |
| *Swimming* | 1 | 0.93% | 0 | 0.00% | 0 | 0.00% | 1 | 2.63% | 1 | 4.55% |
| *Ice Hockey* | 3 | 2.80% | 0 | 0.00% | 1 | 3.45% | 2 | 5.26% | 1 | 4.55% |
| *Other Organized Sports* | 1 | 0.93% | 0 | 0.00% | 0 | 0.00% | 1 | 2.63% | 0 | 0.00% |
| *Dance* | 1 | 0.93% | 1 | 2.50% | 0 | 0.00% | 0 | 0.00% | 0 | 0.00% |
| *Roller Derby* | 1 | 0.93% | 1 | 2.50% | 0 | 0.00% | 0 | 0.00% | 1 | 4.55% |
| *Softball* | 3 | 2.80% | 2 | 5.00% | 0 | 0.00% | 1 | 2.63% | 0 | 0.00% |
| *Biking* | 1 | 0.93% | 0 | 0.00% | 0 | 0.00% | 1 | 2.63% | 1 | 4.55% |
| *BMX* | 1 | 0.93% | 0 | 0.00% | 0 | 0.00% | 1 | 2.63% | 0 | 0.00% |
| Currently Playing (i.e., Is Sport in Season)? | | | | | | | | | | |
| *Yes* | 62 | 57.94% | 24 | 60.00% | 19 | 65.52% | 19 | 50.00% | 4 | 18.18% |
| *No* | 45 | 42.06% | 16 | 40.00% | 10 | 34.48% | 19 | 50.00% | 18 | 81.82% |
| Play Level | | | | | | | | | | |
| *Unknown/Not Reported* | 46 | 42.99% | 16 | 40.00% | 10 | 34.48% | 20 | 52.63% | 18 | 81.82% |
| *College Varsity* | 24 | 22.43% | 12 | 30.00% | 9 | 31.03% | 3 | 7.89% | 2 | 9.09% |
| *Recreational/Intramural* | 13 | 12.15% | 7 | 17.50% | 2 | 6.90% | 4 | 10.53% | 1 | 4.55% |
| *Club/Regional* | 22 | 20.56% | 5 | 12.50% | 8 | 27.59% | 9 | 23.68% | 0 | 0.00% |
| *National/Professional* | 2 | 1.87% | 0 | 0.00% | 0 | 0.00% | 2 | 5.26% | 1 | 4.55% |

**Table S2.** Detailed information about concussions. Note that 4 subjects who did not have a concussion history based on the OSU TBI-ID had 1 diagnosed concussion, and 26 subjects who did have a history of concussion based on the OSU TBI-ID did not have any diagnosed concussion.

| **Concussion & Head Trauma Information (CHx^[[5]](#footnote-5)^a *n* = 67; RHx^[[6]](#footnote-6)^b = 22)** | | | | | |
| --- | --- | --- | --- | --- | --- |
|  | Count | Percent | Average | SE | Range |
| OSU TBI-ID^[[7]](#footnote-7)^c Measure | | | | | |
| *Number of Concussions* | - | - | 2.04 | 0.16 | 1 - 6 |
| *Number of LOC^[[8]](#footnote-8)^d* | 24 | 35.82% | - | - | - |
| *Number of Dazedness/Memory Gap* | 113 | 82.48% | - | - | - |
| *Time Since Last Injury (months;* n *= 66)* | - | - | 56.61 | 6.71 | 1 - 336 |
| *Age at First Injury (years old;* n *= 66)* | - | - | 14.53 | 0.49 | 5 - 24 |
| *Number of Periods of Repetitive Trauma (*n *= 22)* | - | - | 1.23 | 0.13 | 1 - 3 |
| *Age at Onset of First Repetitive Trauma (years old;* n *= 21)* | - | - | 12.91 | 1.19 | 5 - 27 |
| *Duration of First Repetitive Trauma (month;* n *= 20)* | - | - | 6.40 | 1.21 | 0^[[9]](#footnote-9)^e - 21 |
| Cause of Injury | | | | | |
| *Number of Concussive Incidents* | 137 | - | - | - | - |
| *Vehicular Accident* | 8 | 5.84% | - | - | 0 - 1 |
| *Assault* | 1 | 0.73% | - | - | 0 - 1 |
| *Fall, Projectile Hit, or Sports/Playground* | 127 | 92.70% | - | - | 0 - 6 |
| *Explosion or Blast* | 0 | 0.00% | - | - | - |
| *Other/Unknown* | 1 | 0.73% | - | - | 0 - 1 |
| Medical History | | | | | |
| *Number of Diagnosed Concussions* | 94 | - | - | - | 0 - 8 |
| *Number of Reported Hospitalizations* | 21 | - | - | - | 0 - 1 |

**Table S3.** Correlations between different OSU measures, and control, cognitive, and psychological variables. False discovery rate (FDR) corrections for multiple comparisons were performed based on control, cognitive, or psychological variables under analysis, across OSU measures. That is, every *p*-value for a given measure were vectorized such that six values were factored into the correction for that measure, corresponding to the six correlations performed across OSU variables.

| **OSU^[[10]](#footnote-10)^a Metrics & Cognition Correlations** | | | | | |
| --- | --- | --- | --- | --- | --- |
| **Variable** | **N** | ***Spearman’s rho (ρ)*** | ***P (Pcorr)*** | **95% CI** | |
|  |  |  |  | **Lower** | **Upper** |
| **1. Number of Concussions** | | | | | |
| Control Variables | | | | | |
| Age | 107 | .23 | .018 * (.054 .) | 0.04 | 0.40 |
| KBIT^[[11]](#footnote-11)^b (IQ^[[12]](#footnote-12)^c) | 106 | -.10 | .326 (.805) | -0.28 | 0.10 |
| Number of Sports |  | .07 | .466 (.559) | -0.12 | 0.26 |
| Cognitive Variables | | | | | |
| NIH^[[13]](#footnote-13)^d Examiner Battery |  | | | | |
| *Flanker* | 106 | -.20 | .045 * (.270) | -0.37 | -0.01 |
| *2-Back* | 104 | -.12 | .239 (.717) | -0.30 | 0.08 |
| *Set-Shifting* | 107 | -.14 | .144 (.579) | -0.32 | 0.05 |
| Processing Speed |  | | | | |
| *SDMT^[[14]](#footnote-14)^e* | 106 | -.02 | .860 (.992) | -0.21 | 0.17 |
| Hopkins Verbal Learning Test |  | | | | |
| *Trial 1* | 107 | -.10 | .354 (.939) | -0.28 | 0.10 |
| *Trial 2* |  | -.04 | .663 (.888) | -0.23 | 0.15 |
| *Trial 3* |  | -.01 | .937 (.937) | -0.20 | 0.18 |
| *Delay* |  | .05 | .605 (.926) | -0.14 | 0.24 |
| Psychological Variables | | | | | |
| Hospital Anxiety & Depression Scale |  | | | | |
| *Anxiety* | 107 | .15 | .116 (.232) | -0.04 | 0.33 |
| *Depression* |  | .10 | .305 (.462) | -0.09 | 0.29 |
| **2. Number of LOC** | | | | | |
| Control Variables | | | | | |
| Age | 67 | -.03 | .841 (.877) | -0.26 | 0.22 |
| KBIT (IQ) | 66 | .03 | .796 (.805) | -0.21 | 0.27 |
| Number of Sports |  | .12 | .337 (.559) | -0.13 | 0.35 |

| Cognitive Variables | | | | | |
| --- | --- | --- | --- | --- | --- |
| NIH Examiner Battery |  | | | | |
| *Flanker* | 66 | .06 | .622 (.914) | -0.18 | 0.30 |
| *2-Back* | 64 | .22 | .078 . (.468) | -0.03 | 0.44 |
| *Set-Shifting* | 67 | .16 | .193 (.579) | -0.08 | 0.39 |
| Processing Speed |  | | | | |
| *SDMT* | 67 | -.04 | .721 (.992) | -0.28 | 0.20 |
| Hopkins Verbal Learning Test |  | | | | |
| *Trial 1* | 67 | .08 | .546 (.939) | -0.17 | 0.31 |
| *Trial 2* |  | .19 | .127 (.762) | -0.06 | 0.41 |
| *Trial 3* |  | .28 | .025 * (.105) | 0.04 | 0.48 |
| *Delay* |  | .23 | .068 . (.408) | -0.02 | 0.44 |
| Psychological Variables | | | | | |
| Hospital Anxiety & Depression Scale |  | | | | |
| *Anxiety* | 67 | -.14 | .254 (.381) | -0.37 | 0.10 |
| *Depression* |  | -.13 | .308 (.462) | -0.36 | 0.12 |
| **3. Time Since Injury** | | | | | |
| Control Variables | | | | | |
| Age | 66 | .42 | .0005 *** (.003 **) | 0.20 | 0.60 |
| KBIT (IQ) | 65 | .10 | .411 (.805) | -0.14 | 0.34 |
| Number of Sports |  | .10 | .419 (.559) | -0.14 | 0.34 |
| Cognitive Variables | | | | | |
| NIH Examiner Battery |  | | | | |
| *Flanker* | 65 | .03 | .845 (.914) | -0.22 | 0.27 |
| *2-Back* | 63 | -.02 | .875 (.875) | -0.27 | 0.23 |
| *Set-Shifting* | 66 | .11 | .399 (.798) | -0.14 | 0.34 |
| Processing Speed |  | | | | |
| *SDMT* | 66 | -.001 | .992 (.992) | -0.24 | 0.24 |
| Hopkins Verbal Learning Test |  | | | | |
| *Trial 1* | 66 | .09 | .475 (.939) | -0.16 | 0.32 |
| *Trial 2* |  | .04 | .740 (.888) | -0.20 | 0.28 |
| *Trial 3* |  | .12 | .331 (.497) | -0.12 | 0.35 |
| *Delay* |  | -.03 | .830 (.929) | -0.27 | 0.22 |
| Psychological Variables | | | | | |
| Hospital Anxiety & Depression Scale |  | | | | |
| *Anxiety* | 66 | -.28 | .023 * (.138) | -0.49 | -0.04 |
| *Depression* |  | -.20 | .107 (.462) | -0.42 | 0.04 |
| **4. Age at First Injury** | | | | | |
| Control Variables | | | | | |
| Age | 66 | -.02 | .877 (.877) | -0.26 | 0.22 |
| KBIT (IQ) | 65 | .03 | .805 (.805) | -0.21 | 0.27 |
| Number of Sports |  | -.30 | .017 * (.102) | -0.50 | -0.06 |
| Cognitive Variables | | | | | |
| NIH Examiner Battery |  | | | | |

| *Flanker* | 65 | .04 | .763 (.914) | -0.21 | 0.28 |
| --- | --- | --- | --- | --- | --- |
| *2-Back* | 63 | .09 | .478 (.728) | -0.16 | 0.33 |
| *Set-Shifting* | 66 | .01 | .970 (.970) | -0.24 | 0.25 |
| Processing Speed |  | | | | |
| *SDMT* | 66 | -.05 | .694 (.992) | -0.29 | 0.20 |
| Hopkins Verbal Learning Test |  | | | | |
| *Trial 1* | 66 | -.01 | .939 (.939) | -0.25 | 0.23 |
| *Trial 2* |  | -.13 | .302 (.888) | -0.36 | 0.12 |
| *Trial 3* |  | -.26 | .035 * (.105) | -0.47 | -0.02 |
| *Delay* |  | -.06 | .617 (.926) | -0.30 | 0.18 |
| Psychological Variables | | | | | |
| Hospital Anxiety & Depression Scale |  | | | | |
| *Anxiety* | 66 | .23 | .062 . (.186) | -0.01 | 0.45 |
| *Depression* |  | .05 | .719 (.863) | -0.20 | 0.28 |
| **5. Age at Onset of Repetitive Trauma** | | | | | |
| Control Variables | | | | | |
| Age | 21 | .22 | .341 (.512) | -0.24 | 0.59 |
| KBIT (IQ) |  | .07 | .763 (.805) | -0.37 | 0.49 |
| Number of Sports |  | -.19 | .402 (.559) | -0.58 | 0.26 |
| Cognitive Variables | | | | | |
| NIH Examiner Battery |  | | | | |
| *Flanker* | 20 | .03 | .914 (.914) | -0.42 | 0.46 |
| *2-Back* | 21 | .10 | .675 (.810) | -0.35 | 0.51 |
| *Set-Shifting* |  | .11 | .650 (.970) | -0.34 | 0.51 |
| Processing Speed |  | | | | |
| *SDMT* | 21 | .31 | .173 (.992) | -0.14 | 0.65 |
| Hopkins Verbal Learning Test |  | | | | |
| *Trial 1* | 21 | -.03 | .910 (.939) | -0.45 | 0.41 |
| *Trial 2* |  | .11 | .632 (.888) | -0.34 | 0.52 |
| *Trial 3* |  | .33 | .140 (.280) | -0.12 | 0.67 |
| *Delay* |  | .22 | .335 (.926) | -0.23 | 0.60 |
| Psychology Variables | | | | | |
| Hospital Anxiety & Depression Scale |  | | | | |
| *Anxiety* | 21 | .11 | .627 (.627) | -0.34 | 0.52 |
| *Depression* |  | .02 | .931 (.931) | -0.42 | 0.45 |
| **6. Duration of Period of Repetitive Trauma** | | | | | |
| Control Variables | | | | | |
| Age | 20 | .24 | .306 (.512) | -0.23 | 0.62 |
| KBIT (IQ) |  | -.15 | .533 (.805) | -0.55 | 0.32 |
| Number of Sports |  | .06 | .815 (.815) | -0.40 | 0.49 |
| Cognitive Variables | | | | | |
| NIH Examiner Battery |  | | | | |
| *Flanker* | 19 | .09 | .712 (.914) | -0.38 | 0.52 |
| *2-Back* | 20 | -.17 | .485 (.728) | -0.57 | 0.30 |

| *Set-Shifting* |  | -.03 | .907 (.970) | -0.47 | 0.42 |
| --- | --- | --- | --- | --- | --- |
| Processing Speed |  | | | | |
| *SDMT* | 20 | -.03 | .893 (.992) | -0.47 | 0.42 |
| Hopkins Verbal Learning Test |  | | | | |
| *Trial 1* | 20 | -.03 | .894 (.939) | -0.47 | 0.42 |
| *Trial 2* |  | .03 | .890 (.890) | -0.42 | 0.47 |
| *Trial 3* |  | .04 | .858 (.937) | -0.41 | 0.48 |
| *Delay* |  | .02 | .929 (.929) | -0.43 | 0.46 |
| Psychological Variables | | | | | |
| Hospital Anxiety & Depression Scale |  | | | | |
| *Anxiety* | 20 | -.15 | .533 (.627) | -0.55 | 0.32 |
| *Depression* |  | -.27 | .254 (.462) | -0.64 | 0.20 |

Significance flag: . *p* < .10, * *p* < .05, ** *p* < .01, *** *p* < .001

**Table S4.** Summary of pairwise comparison statistics for all metrics under study. Age and the flanker are the only variables for which a Mann-Whitney *U* test is reported due to non-normality of the data. For these two analyses, note that *rrb* is reported (as denoted by italics) as a measure of effect size – not Cohen’s d, as is the case for all other comparisons. False discovery rate (FDR) corrections for multiple comparisons were performed by vectorizing each measure’s (e.g., age, flanker, SDMT, etc.) *p*-values such that its occurrence in each contrast was represented. That is, since there were 3 contrasts, each vector contained 3 *p*-values.

| **Pairwise Comparisons by Group** | | | | | | | | | | |
| --- | --- | --- | --- | --- | --- | --- | --- | --- | --- | --- |
| **Measure** | **N** | **DF** | ***t*** | ***U*** | **Mean**  **Diff.^[[15]](#footnote-15)^a** | **SE** | ***P (Pcorr)*** | ***d/rrb*** | **95% CI** | |
|  |  |  |  |  |  |  |  |  | **Lower** | **Upper** |
| **NCHx^[[16]](#footnote-16)^b vs. RHx^[[17]](#footnote-17)^c** | | | | | | | | | | |
| Control Variables | | | | | | | | | | |
| Age | 57 | - | - | 214.50 | - | - | .017 * (.051 .) | *-.39* | *-.63* | *-.09* |
| KBIT (IQ) |  | 28.51 | 0.91 | - | 3.80 | 4.18 | .371 (.888) | .27 | -.30 | .83 |
| Number of Sports |  | - | - | 367.50 | - | - | .781 (.887) | *.05* | *-.27* | *.36* |
| Cognitive Variables | | | | | | | | | | |
| NIH Examiner  Battery |  | | | | | | | | | |
| *Flanker* | 56 | - | - | 472.00 | - | - | .013 * (.039 *) | *.42* | *.12* | *.66* |
| *2-Back* | 57 | 35.97 | 1.48 | - | 0.29 | 0.19 | .148 (.444) | .42 | -.16 | .98 |
| *Set-Shifting* |  | 31.48 | 1.18 | - | 0.19 | 0.16 | .249 (.669) | .34 | -.23 | .90 |
| Processing Speed |  | | | | | | | | | |
| *SDMT* | 57 | 36.48 | -0.86 | - | -1.90 | 2.22 | .397 (.397) | -.24 | -.80 | .32 |
| Hopkins Verbal  Learning Test |  | | | | | | | | | |
| *Trial 1* | 57 | 30.56 | 0.10 | - | 0.05 | 0.48 | .922 (.922) | .03 | -.53 | .59 |
| *Trial 2* |  | 23.43 | 0.34 | - | 0.18 | 0.52 | .739 (.739) | .10 | -.46 | .66 |
| *Trial 3* |  | 27.67 | 0.33 | - | 0.12 | 0.38 | .746 (.746) | .10 | -.46 | .66 |
| *Delayed Recall* |  | 38.90 | -0.44 | - | -0.21 | 0.49 | .662 (.931) | -.12 | -.68 | .44 |
| Psychological Variables | | | | | | | | | | |
| Hospital Anxiety  & Depression Scale |  | | | | | | | | | |
| *Anxiety* | 57 | 28.55 | -2.29 | - | -2.64 | 1.16 | .030 * (.045 *) | -.67 | -1.25 | -.08 |
| *Depression* |  | 32.95 | -0.44 | - | -0.30 | 0.67 | .663 (.663) | -.13 | -.68 | .44 |
| **SCHx^[[18]](#footnote-18)^d vs. RHx** | | | | | | | | | | |
| Control Variables | | | | | | | | | | |
| Age | 42 | - | - | 152.50 | - | - | .105 (.158) | *-.29* | *-.58* | *.05* |
| KBIT (IQ) | 41 | 32.92 | -0.14 | - | -0.64 | 4.51 | .888 (.888) | -.05 | -.66 | .57 |
| Number of Sports | 42 | - | - | 210.00 | - | - | .887 (.887) | *-.03* | *-.37* | *.32* |
| Cognitive Variables | | | | | | | | | | |
| NIH Examiner Battery |  | | | | | | | | | |
| *Flanker* | 41 | - | - | 252.50 | - | - | .204 (.306) | *.24* | *-.12* | *.54* |
| *2-Back* |  | 37.73 | 0.06 | - | 0.01 | 0.21 | .952 (.952) | .02 | -.60 | .64 |
| *Set-Shifting* | 42 | 38.29 | 0.43 | - | 0.08 | 0.19 | .669 (.669) | .13 | -.48 | .75 |
| Processing Speed |  | | | | | | | | | |
| *SDMT* | 42 | 38.50 | -1.84 | - | -5.86 | 3.09 | .074 . (.222) | -.55 | -1.17 | 0.07 |
| Hopkins Verbal  Learning Test |  | | | | | | | | | |
| *Trial 1* | 42 | 31.13 | -0.71 | - | -0.35 | 0.49 | .482 (.922) | -.23 | -.84 | .39 |

30

| *Trial 2* |  | 35.30 | -0.50 | - | -0.31 | 0.61 | .621 (.739) | -.16 | -.76 | .46 |
| --- | --- | --- | --- | --- | --- | --- | --- | --- | --- | --- |
| *Trial 3* |  | 38.28 | -0.67 | - | -0.31 | 0.46 | .506 (.746) | -.21 | -.82 | .41 |
| *Delayed Recall* |  | 39.98 | -0.76 | - | -0.44 | 0.59 | .454 (.931) | -.23 | -.84 | .38 |
| Psychological Variables | | | | | | | | | | |
| Hospital Anxiety  & Depression Scale |  | | | | | | | | | |
| *Anxiety* | 42 | 35.49 | -1.19 | - | -1.54 | 1.29 | .241 (.254) | -.37 | -.99 | .25 |
| *Depression* |  | - | | | 0.00 | - | | | | |
| **MCHx^[[19]](#footnote-19)^e vs. RHx** | | | | | | | | | | |
| Control Variables | | | | | | | | | | |
| Age | 44 | - | - | 224.50 | - | - | .829 (.829) | *-.04* | *-.37* | *.30* |
| KBIT (IQ) |  | 35.38 | 0.21 | - | 0.98 | 4.62 | .834 (.888) | .07 | -.54 | .67 |
| Number of Sports | 43 | - | - | 273.00 | - | - | .236 (.708) | *.21* | *-.14* | *.52* |
| Cognitive Variables | | | | | | | | | | |
| NIH Examiner  Battery |  | | | | | | | | | |
| *Flanker* | 43 | - | - | 265.00 | - | - | .580 (.580) | *.20* | *-.15* | *.51* |
| *2-Back* | 42 | 38.70 | 0.91 | - | 0.20 | 0.22 | .369 (.554) | .28 | -.34 | .89 |
| *Set-Shifting* | 44 | 40.35 | -0.45 | - | -0.09 | 0.19 | .656 (.669) | -.14 | -.74 | .47 |
| Processing Speed |  | | | | | | | | | |
| *SDMT* | 44 | 41.56 | -0.89 | - | -2.33 | 2.62 | .380 (.397) | -.27 | -.87 | .34 |
| Hopkins Verbal  Learning Test |  | | | | | | | | | |
| *Trial 1* | 44 | 35.95 | -0.45 | - | -0.24 | 0.52 | .654 (.922) | -.14 | -.74 | .46 |
| *Trial 2* |  | 29.90 | -0.53 | - | -0.30 | 0.56 | .599 (.739) | -.17 | -.77 | .44 |
| *Trial 3* |  | 36.94 | -0.34 | - | -0.15 | 0.43 | .739 (.746) | -.10 | -.70 | .50 |
| *Delayed Recall* |  | 36.53 | 0.09 | - | 0.04 | 0.49 | .931 (.931) | .03 | -.57 | .63 |
| Psychological Variables | | | | | | | | | | |
| Hospital Anxiety  & Depression Scale |  | | | | | | | | | |
| *Anxiety* | 44 | 35.68 | -0.79 | - | -1.01 | 1.28 | .435 (.254) | -.24 | -.85 | .36 |
| *Depression* |  | 37.29 | 0.79 | - | 0.58 | 0.73 | .434 (.663) | .24 | -.36 | .84 |

Significance flag: . *p* < .10, * *p* < .05, ** *p* < .01, *** *p* < .001

**Table S5.** These are *post-hoc* contrasts for traditional principal scalars and free-water corrected scalars between our main groups and a subset of the overall sample containing only those who experienced at least one instance of repetitive head trauma (RHx) over the course of their lifetime. The *p*-values were calculated by subtracting the maximum TBSS cluster value from 1. The same multiple corrections scheme as above was used in the table below.

| **TBSS Contrast Results** | | | | | |
| --- | --- | --- | --- | --- | --- |
| **Traditional DTI Scalars** | | | | | |
| Contrast | Comparison | | Modality | Significance Max | *P*-value: 1 – Max (Pcorr) |
| 1, 0, 0, -1 | | NCHx > RHx | FA | .229 | .771 (.807) |
| -1, 0, 0, 1 | | NCHx < RHx |  | .768 | .232 (.696) |
| 0, 1, 0, -1 | | SCHx > RHx |  | .193 | .807 (.807) |
| 0, -1, 0, 1 | | SCHx < RHx |  | .571 | .429 (.807) |
| 0, 0, 1, -1 | | MCHx > RHx |  | .225 | .775 (.807) |
| 0, 0, -1, 1 | | MCHx < RHx |  | .896 | .104 (.624) |
| 1, 0, 0, -1 | | NCHx > RHx | MD | .523 | .477 (.775) |
| -1, 0, 0, 1 | | NCHx < RHx |  | .407 | .593 (.775) |
| 0, 1, 0, -1 | | SCHx > RHx |  | .604 | .396 (.775) |
| 0, -1, 0, 1 | | SCHx < RHx |  | .212 | .788 (.788) |
| 0, 0, 1, -1 | | MCHx > RHx |  | .667 | .333 (.775) |
| 0, 0, -1, 1 | | MCHx < RHx |  | .354 | .646 (.775) |
| 1, 0, 0, -1 | | NCHx > RHx | RD | .444 | .556 (.779) |
| -1, 0, 0, 1 | | NCHx < RHx |  | .252 | .748 (.779) |
| 0, 1, 0, -1 | | SCHx > RHx |  | .593 | .407 (.779) |
| 0, -1, 0, 1 | | SCHx < RHx |  | .221 | .779 (.779) |
| 0, 0, 1, -1 | | MCHx > RHx |  | .684 | .316 (.779) |
| 0, 0, -1, 1 | | MCHx < RHx |  | .256 | .744 (.779) |
| 1, 0, 0, -1 | | NCHx > RHx | AD | .560 | .440 (.640) |
| -1, 0, 0, 1 | | NCHx < RHx |  | .711 | .289 (.640) |
| 0, 1, 0, -1 | | SCHx > RHx |  | .609 | .391 (.640) |
| 0, -1, 0, 1 | | SCHx < RHx |  | .360 | .640 (.640) |
| 0, 0, 1, -1 | | MCHx > RHx |  | .400 | .600 (.640) |
| 0, 0, -1, 1 | | MCHx < RHx |  | .731 | .269 (.640) |
| **Free-Water Imaging Scalars** | | | | | |
| 1, 0, 0, -1 | NCHx > RHx | | FW | .386 | .614 (.737) |

| -1, 0, 0, 1 | NCHx < RHx |  | .574 | .426 (.737) |
| --- | --- | --- | --- | --- |
| 0, 1, 0, -1 | SCHx > RHx |  | .850 | .150 (.737) |
| 0, -1, 0, 1 | SCHx < RHx |  | .144 | .856 (.856) |
| 0, 0, 1, -1 | MCHx > RHx |  | .499 | .501 (.737) |
| 0, 0, -1, 1 | MCHx < RHx |  | .488 | .512 (.737) |
| 1, 0, 0, -1 | NCHx > RHx | FAt | .241 | .759 (.759) |
| -1, 0, 0, 1 | NCHx < RHx |  | .680 | .320 (.759) |
| 0, 1, 0, -1 | SCHx > RHx |  | .296 | .704 (.759) |
| 0, -1, 0, 1 | SCHx < RHx |  | .302 | .698 (.759) |
| 0, 0, 1, -1 | MCHx > RHx |  | .301 | .699 (.759) |
| 0, 0, -1, 1 | MCHx < RHx |  | .931 | .069 (.414) |
| 1, 0, 0, -1 | NCHx > RHx | MDt | .721 | .279 (.712) |
| -1, 0, 0, 1 | NCHx < RHx |  | .181 | .819 (.831) |
| 0, 1, 0, -1 | SCHx > RHx |  | .335 | .665 (.831) |
| 0, -1, 0, 1 | SCHx < RHx |  | .682 | .318 (.712) |
| 0, 0, 1, -1 | MCHx > RHx |  | .644 | .356 (.712) |
| 0, 0, -1, 1 | MCHx < RHx |  | .169 | .831 (.831) |
| 1, 0, 0, -1 | NCHx > RHx | RDt | .535 | .465 (.858) |
| -1, 0, 0, 1 | NCHx < RHx |  | .222 | .778 (.858) |
| 0, 1, 0, -1 | SCHx > RHx |  | .288 | .712 (.858) |
| 0, -1, 0, 1 | SCHx < RHx |  | .551 | .449 (.858) |
| 0, 0, 1, -1 | MCHx > RHx |  | .766 | .234 (.858) |
| 0, 0, -1, 1 | MCHx < RHx |  | .142 | .858 (.858) |
| 1, 0, 0, -1 | NCHx > RHx | ADt | .760 | .240 (.494) |
| -1, 0, 0, 1 | NCHx < RHx |  | .527 | .473 (.494) |
| 0, 1, 0, -1 | SCHx > RHx |  | .506 | .494 (.494) |
| 0, -1, 0, 1 | SCHx < RHx |  | .730 | .270 (.494) |
| 0, 0, 1, -1 | MCHx > RHx |  | .557 | .443 (.494) |
| 0, 0, -1, 1 | MCHx < RHx |  | .630 | .370 (.494) |

1. a No concussion history [↑](#footnote-ref-1)
2. b Single concussion history [↑](#footnote-ref-2)
3. c Multiple concussion history [↑](#footnote-ref-3)
4. d Repeated head-trauma history [↑](#footnote-ref-4)
5. a CHx is used to denote those with some concussion history [↑](#footnote-ref-5)
6. b RHx includes those who experienced at least one extended period of time where they sustained regular-subclinical impacts to the head. This group is a subset of the total sample and includes those who have and have not sustained at least one or more concussions. Note that while 22 people meet criteria for belonging to this group, two people were missing data for duration of trauma (i.e., dates of onset and cessation were required to make this calculation), and one person was missing data for age of onset. [↑](#footnote-ref-6)
7. c Ohio State University Traumatic Brain Injury-Identification [↑](#footnote-ref-7)
8. d Losses of consciousness [↑](#footnote-ref-8)
9. e The minimum of 0 indicates that the period of repetitive head trauma began and ended within the same 1-month period. [↑](#footnote-ref-9)
10. a Ohio State University [↑](#footnote-ref-10)
11. b Kauffman Brief Intelligence Test [↑](#footnote-ref-11)
12. c Intelligence Quotient [↑](#footnote-ref-12)
13. d National Institute of Health [↑](#footnote-ref-13)
14. e Symbol-Digit Modalities Test [↑](#footnote-ref-14)
15. a Difference [↑](#footnote-ref-15)
16. b No concussion history [↑](#footnote-ref-16)
17. c History of subclinical repetitive trauma [↑](#footnote-ref-17)
18. d Single concussion history [↑](#footnote-ref-18)
19. e Multiple concussion history [↑](#footnote-ref-19)
